# The Reliability of Specific Physical Fitness Assessments in Elite Female Chinese Wrestlers

**DOI:** 10.5114/jhk/187855

**Published:** 2024-07-17

**Authors:** Yinchuan Bai, Naidan Xu, Xiangchen Li, Yupeng Shen

**Affiliations:** 1 Smart Sports and Innovation Research Centre, China Institute of Sport Science, Beijing, China.; 2 Sports Academy, Beijing Sport University, Beijing, China.; 3 School of Physical Education and Sports Science, South China Normal University, Guangzhou, Guangdong, China.

**Keywords:** combat sports, specific tests, coordination, speed, strength and endurance, consistency

## Abstract

The aim of this study was to evaluate the reliability of eight specific fitness tests for elite female Chinese wrestlers. Twenty-eight elite female wrestlers participated in the study (age: 26.9 ± 2.81 years). The reliability of the tests was analyzed using the intraclass correlation coefficient (ICC) and the 95% confidence interval (CI), the coefficient of variation (CV), and other metrics. The 30-s Sit-Up (SU30) and 6-m Rope Climb (RC6m) tests showed excellent reliability (ICC > 0.9). The 30-s Dummy Throw (DT30) had good to excellent reliability, while the 30-s Bridge-Return (B-R30) showed moderate to good reliability. The 30-s Burpee (BUR30), 15-s Leg Attack (LA15), 15-s Leg Defense (LD15), and Dummy Suplex and Gut Wrench (DS&GW) tests ranged from poor to good reliability. SU30, DT30, LA15, and RC6m tests displayed low variability (CV < 5%), while others exhibited moderate variability. SU30, B-R30, DT30, and RC6m tests are reliable for assessing wrestling fitness. However, BUR30 and LA15 tests showed high variability and should be used carefully. LD15 and DS&GW tests are not recommended for assessing fitness in elite female wrestlers.

## Introduction

Wrestling can be traced back to the ancient Greek Olympics and is one of the combat sports included in the Summer Olympics (Zaccagni, 2012). In wrestling, athletes need to possess high levels of strength, speed, muscular endurance, and isometric strength to execute various technical moves (Scott et al., 1990). Regarding the time structure of wrestling matches, medalists or gold medalists typically need to complete 5 to 6 matches in one day, with each round consisting of two bouts, each bout lasting 3 min, with a 30-s rest interval between the two bouts. This undoubtedly places high demands on the physical fitness of wrestlers. Studies have suggested that a high-level wrestler must have strong physical fitness to attack opponents within 20–25 s of working time throughout the entire match, especially when fatigued or in a passive position, and to continue effectively using technical moves to score or win (Podlivaev, 2015).

Physical fitness can be divided into general fitness and specific fitness. General fitness reflects the athlete's basic physical abilities and is a comprehensive manifestation of the athlete's physical qualities. In contrast, specific fitness refers to the morphology, physiological functions, and physical qualities closely related to a specific sport. Physical qualities are the core elements of specific fitness, which directly promotes the mastery of specific techniques and the improvement of specific sports performance. In wrestling training and competition, there are many factors that influence a wrestler's specific fitness level. However, specific anaerobic capacity and specific physical qualities are considered the most crucial elements. Numerous studies have shown that anaerobic capacity is a key factor for determining the result of wrestling competitions (Horswill,1992; Horswill et al., 1989; Pallarés et al., 2012). Additionally, specific physical fitness, which mainly includes specific coordination, speed, strength, along with specific strength and endurance, play a decisive role in specific physical fitness (Podlivaev, 2015).

Various fitness tests are widely used for assessing the physical performance of combat athletes. However, most studies have focused on evaluating the general physical fitness (physiological and physical qualities) of athletes in combat sports (amateur boxing, fencing, judo, karate, taekwondo and wrestling) rather than specific physical fitness (Bridge et al., 2014; Chaabene et al., 2015, 2015a, 2017a; Franchini et al., 2011; Kuvačić et al., 2017; Roi et al., 2008). In contrast, sport-specific tests can help coaches identify promising young athletes, pinpointing their strengths and weaknesses in terms of physical qualities and physiology during training. Additionally, sport-specific test results can also help coaches monitor how athletes' performance changes and develops over time (Tabben et al., 2014). Therefore, establishing effective sport-specific testing protocols to evaluate the actual physical fitness and physiological characteristics of combat athletes is crucial for the advancement of sports science.

An increasing number of researchers have begun to develop effective combat sports-specific testing protocols that aim to meet the key requirements of various combat sports in terms of physical, physiological, technical, and tactical aspects (Chaabène et al., 2012; Marković et al., 2022; Sant'Ana et al., 2019; Santos et al., 2010; Tabben et al., 2014). However, research dedicated to developing sport-specific performance assessments tailored to the physical demands of wrestlers is sparse (Klinzing et al., 1983; Utter et al., 1997). A few studies have focused on specific anaerobic capacity (Marković et al., 2021; Shiyan, 2011; Utter et al., 1997; Wright et al., 2015). Researchers have validated the reliability and efficacy of these wrestling-specific anaerobic capacity testing protocols, deeming the existing protocols suitable for standardized application (Marković et al., 2021). However, there is still a lack of research on effective and reliable tools for assessing specific physical qualities that can be used for large-scale wrestling training.

Therefore, this study aimed to evaluate the reliability of commonly used specific physical fitness testing protocols for elite Chinese female wrestlers using a repeated-measures design, analyze the test-retest variability and consistency of various specific physical fitness testing protocols, and ultimately establish a standardized specific physical fitness testing protocol for elite female wrestlers.

## Methods

### Participants

Twenty-eight elite female wrestlers participated in the study (mean ± SD: age 26.9 ± 2.81 years, body height 165 ± 5.66 cm, body mass [BM] 67.0 ± 9.04 kg, body mass index [BMI] 24.4 ± 2.12%, and training experience 12.6 ± 2.04 year). The included wrestlers did not have injuries of the upper limbs, lower limbs, the torso, the head or the neck, and none had undergone surgery on any body part within the last six months. Among the wrestlers, 50% (14 athletes) had won medals in the World Wrestling Championships, while the remaining athletes were medalists in the National Wrestling Championships. All wrestlers voluntarily participated in this study. Both wrestlers and coaches were informed of the potential risks and benefits of the study which was conducted following the principles of the Declaration of Helsinki, and approved by the Ethics Committee of the China Institute of Sport Science (protocol code: CISSLA-2022-1104; approval date: 04 November 2022). All participants provided written informed consent before the commencement of the study.

### Measures

#### 30-s Bridge-Return Test

The B-R30 primarily evaluates the strength and coordination of the shoulder and neck muscles, as well as the flexibility and coordination of the neck, the waist, the back, and hips of wrestlers. The B-R30 procedure is as follows: participants start from the bridge position, using their head as the axis and both hands on either side of the head for support. At the start of the movement, athletes push off the ground with their legs, extend their hips, raise their head, and swing forward to perform a front bridge swing. Once the body passes the vertical plane, participants need to tighten their abdomen and land on both feet, entering a headstand. Then, they swing backward into the bridge position. Completion of this series of movements constitutes one bridge-return action. During the test, participants need to complete the bridge-return action as quickly as possible, and the maximum number of repetitions within 30 s is recorded.

#### 30-s Burpee Test

The BUR30 mainly evaluates the specific coordination and fluid motions of wrestlers moving from the standing position to the par-terre position. The BUR30 procedure is as follows: athletes start the action from a standing position with their feet shoulder-width apart. At the start of the movement, they quickly squat and place their hands on the ground while extending their feet backwards, straightening their bodies and contacting the ground, then they enter the plank position. Next, as athletes support themselves with their hands on the ground, they jump with their feet forward to the outside of their hands, entering a squat position, and jump vertically upwards. After their feet touch the ground, one burpee movement is completed. During the test, athletes need to complete burpee movements as quickly as possible, and the maximum number of repetitions completed within 30 s is recorded.

#### 6-m Rope Climb without Legs

The RC6m mainly evaluates the specific strength and endurance of the upper limbs and the grip strength of wrestlers. The RC6m procedure is as follows: athletes start from a standing position facing the training rope, choose an appropriate height, and grip the rope with both hands. At the start of the movement, athletes use their arms to pull themselves upward, and with the help of inertia, they alternate hands as they rapidly shift their grip upward, continually pulling themselves upward without using their legs. The test is considered complete when either hand touches the 6-m mark on the training rope, and the corresponding time is recorded. Additionally, during the descent, athletes must use both their hands and legs to return to the ground. Jumping directly of the rope is prohibited.

#### 30-s Sit-Up Test

The SU30 mainly evaluates the specific strength and endurance of the core muscles of wrestlers and also assesses the ability of athletes to perform technical moves repeatedly over a prolonged period. The SU30 procedure is as follows: athletes start from a supine position on an inclined board, with their feet higher than their head, knees bent, and their feet hooked on the straps of the inclined board, with their hands crossed in front of their chest. At the beginning of the movement, using the hip joint as the axis, the athlete actively contracts the core muscles to flex the upper body toward the knees, trying to touch the chin to the knees with each flexion. Then, the athlete returns backward in a controlled manner, lightly touching the back to the inclined board. This series of movements counts as one sit-up, and athletes are instructed not to let the back completely touch the inclined board. The entire test consists of three sets, each lasting 30 s, with a 10-s rest interval in between. During the test, athletes must complete the sit-ups as quickly as possible, and the maximum number of repetitions completed in each set is recorded. The sum of the number of repetitions in the three sets equals the final score.

#### 15-s Leg Attack Test (without a Partner)

The LA15 mainly evaluates the speed of wrestlers when performing leg attacks. The LA15 procedure involves the following steps: athletes start by standing with their feet parallel or with one foot in front of another, assuming a wrestling-specific body posture. At the start of the movement, athletes quickly perform a single-leg attack to the left or the right side. During the test, athletes need to quickly lower their center of gravity, perform a duck-under movement, step forward with the leading leg, actively extend the arm on the same side forward, and push off with the trailing leg while the torso quickly follows. Then, athletes quickly return to the wrestling position, completing one leg attack movement. During the test, athletes need to complete as many leg attack movements as possible within 15 s, and the total number of repetitions is recorded.

#### 15-s Leg Defense Test (without a Partner)

The LD15 evaluates the speed of wrestlers when performing a leg defense. The LD15 procedure is as follows: athletes start by standing with their feet parallel or with one foot in front of another, entering a specific wrestling posture. At the start of the movement, upon hearing the whistle, athletes imagine that they are targeted for leg takedown by an opponent and swiftly react by executing a leg defense. During the test, athletes need to quickly lower their center of gravity, place both hands on the ground, extend their legs backward, and try to make their hip joints move as close to the ground as possible. Then, athletes quickly return to the wrestling posture, completing one leg defense movement. During the test, athletes need to complete the leg defense movement as quickly as possible, and the total number of repetitions completed within 15 s is recorded.

#### 30-s Dummy Throw Test

The DT30 mainly evaluates the specific speed strength and endurance of wrestlers' technical movements. The DT30 procedure is as follows: athletes start from a standing position facing the dummy. At the start of the movement, they lower quickly their center of gravity, step forward and hold the dummy's shoulder or waist using a back throw technique, quickly rotate their body, and perform a throwing action to the left or the right side. Each time the dummy is thrown to the ground, it counts as one dummy throw movement. During the test, athletes complete the dummy throw movement as quickly as possible, and the total number of repetitions completed within 30 s is recorded.

#### Dummy Suplex and Gut Wrench Test

The DS&GW mainly evaluates the specific speed, strength and endurance of wrestlers' technical movements while moving from the standing to the par-terre position. The DS&GW involves the following steps: athletes start in a standing position facing the dummy. At the start of the movement, they lower quickly their center of gravity and step forward to grab the dummy's waist or chest. Using a suplex technique, athletes quickly push off the ground, extend their hips, rotate their body to the left or right, and perform the dummy suplex maneuver. Once the dummy lands on the mat, athletes should rapidly grasp the dummy’s waist or chest and use a gut wrench technique. With the upper body tightly holding the dummy, athletes lower their limbs, push off the ground, extend the hips, and roll the dummy to the left or the right side. The completion of the gut wrench technique marks the end of one DS&GW movement. The entire test consists of four DS&GW combinations, with each complete DS&GW including one suplex and one gut wrench maneuver. During the test, athletes are required to complete the transition from standing to the par-terre position as quickly as possible, and the corresponding time to complete the entire test is recorded.

### Design and Procedures

#### Design

A repeated-measures design was used to evaluate the reliability of testing protocols for evaluating the specific physical fitness of elite female wrestlers and to provide reliable standardized fitness testing and training tools for elite female wrestlers. We selected eight commonly used specific physical fitness testing protocols to assess their suitability for elite female wrestlers. The testing protocols referred to the work-to-rest ratio in wrestling matches (2.5:1 or 3:1), the structure of matches (duration), and the specific technical movements. We focused on specific coordination, speed, strength and endurance, which play decisive roles in wrestling performance. Each test represents a specific physical ability of athletes, as shown in Table 1.

**Table 1:** Characteristics of specific physical fitness tests.

Test name (abbreviation)	Physical attribute(s) tested	Recorded variables
***30-s Bridge-Return (B-R30)***	coordination and strength of the neck and the shoulders	coordination and strength of the neck, the trunk and the lower body
***30-s Burpee (BUR30)***	coordination	explosive coordination of wrestlers moving from standing to par-terre positions
***6-m Rope Climb without Legs (RC6m)***	specific strength	specific strength and endurance of the upper limbs
***30-s Sit-Up (SU30)***	strength endurance	endurance of core muscles
***15-s Leg Attack (LA15)***	specific speed	speed of a leg attack
***15-s Leg Defense (LD15)***	specific speed	speed of a leg defense
***30-s Dummy Throw (DT30)***	speed and endurance	specific endurance of technical movements
***Dummy Suplex and Gut Wrench (DS&GW)***	specific endurance	specific endurance of technical movements from standing to par-terre positions

To achieve effective evaluation, we selected testing indicators that were as consistent as possible with the working duration of wrestling matches and similar to the combination of specific technical movements utilized. During the testing process, we used a dummy with uniform height and standardized weight as a substitute for a testing partner. The height of the dummy was 1.6 m, the dummy weight selection was aligned with the women's competition categories and their daily body weights. Athletes weighing less than 55 kg used a 30-kg dummy, those between 62 and 68 kg used a 40-kg dummy, and those between 74 and 82 kg used a 50-kg dummy. The chosen dummy weight represented about 55% of the athlete's body weight, ensuring that the relative load of the dummy was equalized between the subjects relative to their competing category. The testing indicators used in the study are commonly used, thus eliminating the impact of learning on the results (i.e., athletes were already familiar with the general movements). Additionally, we unified the order of the tests to eliminate the influence of the testing sequence on the results. All these measures were taken to minimize testing errors to the possible extent.

#### Procedures

**Figure 1:**
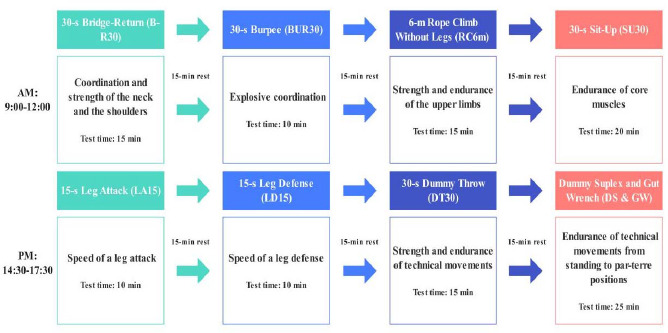
Order and timing of the specific physical fitness assessments.

All athletes completed the tests on the same day, with four tests performed in the morning and four tests in the afternoon, as shown in Figure 1. The morning tests and their order were as follows: 30-s Bridge-Return (B-R30), 30-s Burpee (BUR30), 6-m Rope Climb without Legs (RC6m), and 30-s Sit-Up (SU30). The afternoon tests and their order were as follows: 15-s Leg Attack (LA15) , 15-s Leg Defense (LD15) , 30-s Dummy Throw (DT30), Dummy Suplex and Gut Wrench (DS&GW). All tests were conducted in the wrestling venue at the Chinese National Olympic Training Center which was fully equipped and suitable for multiple testing at the same time. Before testing, physical trainers prepared the test field and equipment according to the group of subjects. Twenty-eight participants were randomly divided into four groups of seven, and each group was evaluated by one national-level wrestling coach and one judge, and each group was tested simultaneously according to the test content and order. To ensure the accuracy of counting and timing, all testing procedures were recorded on video. In the event of any discrepancies during the test, video review was used.

The complete testing process and content are illustrated in Figure 1. The tests were divided into morning and afternoon sessions, each part consisting of four tests. Each test lasted 10 to 20 min and total test time was about 1 h. There was a 15-min rest interval after each test and the total rest time was 1 h. The testing time of each part was approximately 2 h. The morning tests focused on neck and shoulder strength, coordination, explosive power, upper limb strength and abdominal muscle endurance, while the afternoon tests concentrated on technical movements and specific physical fitness tests performed with the use of a dummy. Each test targeted different body parts and skills, ensuring ample rest to minimize fatigue.

The retest interval was one week, and retests were conducted under the same conditions as the initial tests. This decision was based on multiple studies in the reliability of specialized physical fitness testing in combat sports, which have demonstrated good reliability when retesting is conducted within a week (Chaabène et al., 2018; Rocha et al., 2016; Santos et al., 2010; Tabben et al., 2014). Participants were instructed not to engage in vigorous exercise 24 h before testing, and there were no other sports activities on the day of the test. Additionally, athletes completed a standardized warm-up before testing.

### Statistical Analysis

Data are described with the mean and standard deviation. The Shapiro-Wilk test was used to assess the normality of data distribution, and all variables conformed to a normal distribution. It was important to assess the change in means rather than within-participant variability and test-retest correlations (Hopkins, 2000). Therefore, paired sample *t*-tests were used to compare scores on the two tests, with effect size statistics calculated to estimate the degree of difference between the tests; the magnitude of the effect was interpreted as trivial (<0.2), small (0.2–0.6), moderate (>0.6–1.2), large (>1.2–1.99), or very large (≥2.0) (Zimmerman, 1997).

The test-retest reliability of each variable was assessed by the intraclass correlation coefficient (ICC) using a two-way, random, single-measure model, absolute error (AE), typical error (TE), the coefficient of variation (CV), the standard error of measurement (SEM), and the minimal detectable change (MDC) based on a 95% confidence interval (CI). ICC values were interpreted as poor (< 0.5), moderate (0.5–0.75), good (0.75–0.9) or excellent (> 0.9) reliability. CV values were interpreted as good (< 5%), moderate (5–10%) or poor (> 10%). SEM values less than, similar to, or greater than the MDC were rated as "good", "acceptable" or "marginal", respectively (Buchheit et al., 2011).

Bland-Altman plots were used to display within-group variation and systematic changes between tests: bias (mean difference) and the upper (ULoA) and lower (LLoA) limits of agreement were computed (Bland, 1986). Statistical analysis and plotting were performed using the R programming language (version 4.0.2) with the psych, blandr, ggplot, rstatix, and irr packages.

## Results

### Comparison of Test-Retest Results regarding Assessments of Specific Physical Fitness

Table 2 shows the average scores and standard deviations (mean ± SD) as well as the effect sizes (*d*) of B-R30, BUR30, RC6m, SU30, LA15, LD15, DT30 and DS&GW at test and retest. The results of paired sample *t*-tests indicated that B-R30 (*d* = 0.36), BUR30 (*d* = 0.45), RC6m (*d* = 0.34), SU30 (*d* = 0.20), LA15 (*d* = 0.51) and DT30 (*d* = 0.30) scores exhibited only minor differences between test and retest. LD15 (*d* = 0.94) scores showed a moderate difference between test and retest, while DS&GW (*d* = −1.35) scores exhibited a larger difference.

**Table 2:** Comparison of test-retest results regarding assessments of specific physical fitness.

Test name	Test score	Retest score	Diff [95% CI]	t	Cohen’s d	Effect size magnitude
***B-R30 (n)***	11.6 ± 2	11.1 ± 2.4	0.5 [−0.03, 1.03]	1.93	0.36	Small
***BUR30 (n)***	9 ± 1.7	8.46 ± 1.7	0.54 [0.07, 1]	2.36	0.45	Small
***RC6m (s)***	10.2 ± 1.59	10.1 ± 1.57	0.07 [−0.01, 0.15]	1.78	0.34	Small
***SU30 (n)***	63.0 ± 7.2	62.6 ± 6.7	0.39 [−0.36, 1.15]	1.06	0.20	Small
***LA15 (n)***	5.4 ± 0.6	5.3 ± 0.5	0.21 [0.05, 0.38]	2.71	0.51	Small
***LD15 (n)***	5.3 ± 0.6	4.7 ± 0. 7	0.68 [0.4, 0.96]	4.97	0.94	Moderate
***DT30 (n)***	9.9 ± 1.7	9.6 ± 1.6	0.21 [−0.05, 0.48]	1.65	0.30	Small
***DS&GW (s)***	52.6 ± 6.7	56.8 ± 6.1	−4.14[−5.33, −2.95]	−7.16	−1.35	Large

### Test-Retest Reliability Results regarding Assessments of Specific Physical Fitness

Table 3 shows the test-retest reliability values of B-R30, BUR30, RC6m, SU30, LA15, LD15, DT30 and DS&GW according to test-retest comparisons, including values of the absolute difference (AE), CV, SEM, MDC, and ICC.

**Table 3:** Test-retest reliability results regarding assessments of specific physical fitness.

Test name	AE	TE	CV	SEM	MDC	ICC	95% CI lower	95%CI upper
***B-R30 (n)***	1.1	0.57	7.14	0.19	0.16	0.79	0.59	0.90
***BUR30 (n)***	0.96	0.48	7.81	0.17	0.14	0.70	0.42	0.85
***RC6m (s)***	0.11	0.056	0.79	0.03	0.02	0.99	0.98	0.99
***SU30 (n)***	1.6	0.80	1.81	0.26	0.23	0.96	0.92	0.98
***LA15 (n)***	0.21	0.11	2.83	0.06	0.03	0.70	0.41	0.86
***LD15 (n)***	0.7	0.34	9.56	0.1	0.1	0.25	−0.08	0.56
***DT30 (n)***	0.5	0.25	3.63	0.09	0.07	0.91	0.81	0.96
***DS&GW (s)***	4.14	2.07	5.36	0.68	0.59	0.74	−0.03	0.92

* AE = absolute difference; CV = coefficient of variation; SEM = standard error of the mean; MDC = minimum detectable change based on a 95% confidence interval; ICC = intraclass correlation coefficient

The data showed that, except for DS&GW (AE = 4.14), AE values of the other variables were relatively low (AE = 0.11–1.6). RC6m and SU30 tests had both excellent consistency, with 95% CIs of ICCs of 0.98–0.99 and 0.92–0.98, respectively. The DT30 had good to excellent reliability (95% CI of ICC = 0.81–0.96), while the B-R30 had moderate to good reliability (95% CI of ICC = 0.59–0.90). BUR30, LA15, LD15 and DS&GW tests had poor to good reliability (95% CIs of ICCs = −0.08–0.92). SU30, DT30, LA15, and RC6m tests presented good levels of variability (CV < 5%), while B-R30, BUR30, LD15 and DS&GW tests were characterized by moderate levels of variability (CV = 5.36%–9.56%). The SEM for all variables was slightly larger than the MDC.

**Figure 2:**
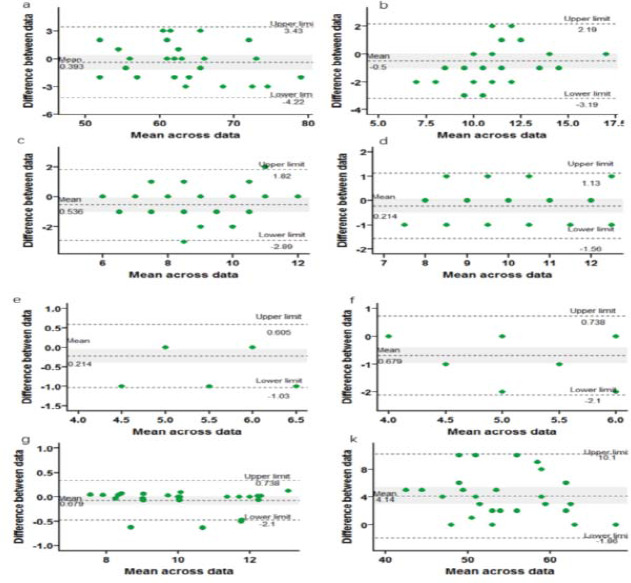
Bland-Altmann plots of the test-retest difference (y-axis) and the mean of the measurements (x-axis).

Figure 2 is an Bland-Altmann plot showing the test-retest differences (y-axis) and the mean scores (x-axis). The mean scores and test-retest differences of the assessments were as follows: SU30 (bias = 0.39, 95% CI = −3.43–4.21), B-R30 (bias = 0.5, 95% CI = −2.19–3.19), DT30 and B-R30 (bias = 0.54, 95% CI = −1.82–2.89), LA15 (bias = 0.21, 95% CI = −0.6–1.03), LD15 (bias = 0.68, 95% CI = −0.7–2.1) and DS&GW (bias = −4.14, 95% CI = −10.15–1.86). All these assessments had bias values within the 95% confidence interval. However, BUR30 (bias = 0.54, 95% CI = −1.82–2.89) and RC6m (bias = 0.07, 95% CI = −0.34–0.48) tests had 3% (1/28) and 7% (2/28) of the data outside the 95% confidence interval, respectively.

## Discussion

This is the first study to evaluate the test-retest reliability of eight commonly used specific physical fitness tests in elite Chinese female wrestlers. B-R30, SU30 and DT30 tests demonstrated good levels of consistency. The B-R30 reflected the wrestlers' specific coordination (Gierczuk et al., 2012), while the SU30 evaluated their core strength and endurance (Cordo et al., 2003). Both tests involved a single cyclical movement structure (Cordo et al., 2003; Kerr et al., 1997). Throughout the SU30, athletes maintained fixed points of support and demonstrated consistent technical movements, not influenced by the testing tools or partners, which may be the main explanation for the high consistency observed in consecutive assessments of B-R30 and SU30 tests.

On the other hand, the DT30 primarily reflected the wrestlers' specific strength and endurance (Bland, 1986). The technical movement transitions were relatively stable, and the wrestler used highly familiar throwing techniques during the test. Additionally, the test dummy used by athletes was highly consistent and standardized in weight. As a result, within the stipulated time frame, errors in the DT30 were effectively controlled, resulting in a high level of consistency.

The RC6m reflected the specific strength and endurance of the upper limbs and the hand grip strength of wrestlers (Baláš et al., 2012). Previous studies have shown that grip strength and upper limb strength display both high levels of consistency, which may be the main explanation for the high reliability of RC6m scores. Although in this study, 6% of RC6m scores were outside the 95% CI of the MDC in the Bland-Altman plot, the bias level in the reliability results (bias = 0.07, 95% CI = −0.34–0.48) was less than 0.1 s. In practical training terms, minor errors had almost no impact on the test results. Hence, the RC6m also exhibited a good level of reliability and can be used as a training tool by coaches.

The test-retest differences of BUR30 and LA15 tests were relatively large, thus coaches should use these assessments cautiously. The BUR30 evaluated the specific coordination and fluid motions of wrestlers transitioning from standing to the par-terre position. Although the BUR30 involved a fixed combination of movements, these actions were relatively complex, including standing, squatting, push-ups, and vertical jumps (Tai et al., 2022). The proficiency and continuity of these movements may lead to changes in ICC values. However, the BUR30 can accurately simulate specific wrestling skills and is not affected by external factors (such as the testing tools or partners); thus, it can still be used as an assessment tool as long as the athlete is highly proficient in those movements. The LA15 assessed the speed of technical wrestling movements. It covered continuous movements such as quick reactions, double foot starts, changes in body weight distribution, and leg grabbing techniques (Jones, 2012), which may explain the variations in ICC values. However, leg attacks are the most commonly used scoring technique in wrestling and a key technique for standing training (Cipriano, 1993). Additionally, researchers believe that this assessment is still useful, provided that it is standardized and familiar to athletes.

LD15 and DS&GW tests are commonly used by coaches to assess the specific physical fitness levels of elite female wrestlers. However, in this study, LD15 and DS&GW tests showed a large variation in ICC values, suggesting low consistency. They are not recommended as tools for evaluating the specific physical fitness of elite female wrestlers, which is the most important conclusion of this study. One possible explanation is that both tests place a high demand on lower limb techniques and wrestler strength. Research has shown that the reliability of lower limb strength tests is low (Tabor et al., 2002). In addition, although the movement structure of both tests is fixed and not affected by external factors during the test, the combination of movements and technical requirements for both tests is relatively complex. For example, the LD15 requires athletes to quickly respond to simulated situations where they are attacked by the opponent's legs, involving a series of complex techniques such as quick reactions, double foot starts and double leg retractions. The DS&GW movements are even more complex, involving more technical movement combinations in both standing and par-terre positions. The complexity, speed of movement transitions and variation in athletes' training may explain the low reliability values. Therefore, we do not recommend using these two assessments to evaluate the specific physical fitness of elite female wrestlers and believe that in practical training contexts, there may be significant variations in athletes' performance between tests. Additionally, these variations may not accurately reflect the true differences in specific physical fitness levels.

This study is not without limitations. The applied tests were not conducted in random order, and although reliability was assessed, further research with larger sample sizes should be conducted due to the diversity of wrestlers. Additionally, male wrestlers and non-elite athletes were not included in the study. Therefore, future testing and validation should be conducted with samples including male wrestlers and non-elite athletes.

## Conclusions

As shown above, SU30, B-R30, DT30 and RC6m tests had good reliability and can be used as important assessment tools for evaluating specific physical fitness of elite female wrestlers. However, coaches and athletes should use BUR30 and LA15 tests with caution due to the high variability in scores of these assessments. LD15 and DS&GW tests had poor reliability and are not recommended for use as assessment tools to monitor training of elite female wrestlers.
